# Nocebo hyperalgesia can be induced by classical conditioning without involvement of expectancy

**DOI:** 10.1371/journal.pone.0232108

**Published:** 2020-05-07

**Authors:** Elżbieta A. Bajcar, Wacław M. Adamczyk, Karolina Wiercioch-Kuzianik, Przemysław Bąbel

**Affiliations:** 1 Pain Research Group, Institute of Psychology, Jagiellonian University, Kraków, Poland; 2 Department of Kinesiotherapy and Special Methods in Physiotherapy, The Jerzy Kukuczka Academy of Physical Education, Katowice, Poland; University of Modena and Reggio Emilia, ITALY

## Abstract

Influential theoretical accounts take the position that classical conditioning can induce placebo effects through conscious expectancies. In the current study two different conditioning procedures (hidden and open) were used to separate expectancy from conditioning in order to reveal the role of expectancy in the formation of nocebo hyperalgesia. Eighty-seven healthy females were randomly assigned to three groups (hidden conditioning, open conditioning, and control). Participants were selected according to the Fear of Pain Questionnaire scores and assigned to two subgroups: high and low level of fear of pain (trait). They received electrocutaneous pain stimuli preceded by either an orange or blue color. During the conditioning phase, one color was paired with pain stimuli of moderate intensity (control stimuli) and the other color was paired with pain stimuli of high intensity (nocebo stimuli) in both hidden and open conditioning groups. Only participants in the open conditioning group were informed about this association, however just before the testing phase the expectancy of hyperalgesia induced in this way was withdrawn. In the control group, both colors were followed by control pain stimuli. During the testing phase all participants received a series of stimuli of the same intensity, regardless of the preceding color. Participants rated pain intensity, expectancy of pain intensity and fear (state). We found that nocebo hyperalgesia was induced by hidden rather than open conditioning. The hidden conditioning procedure did not produce conscious expectancies related to pain. Nocebo hyperalgesia was induced in participants with low and high fear of pain and there was no difference in the magnitude of the nocebo effect between both groups. Nocebo hyperalgesia was not predicted by the fear of upcoming painful stimuli.

## Introduction

Recent years have seen advances in placebo effect research, but less attention has been paid to the nocebo effect [1,2]. The term “nocebo effect” was originally coined to describe noxious or undesirable effects caused by the use of placebo [3,4], and this way of defining the nocebo effect remains valid today [5]. However, two phenomena must be distinguished: the placebo and nocebo effect vs the placebo and nocebo response. The placebo and nocebo effect refers to "… the changes specifically attributable to placebo and nocebo mechanisms, including the neurobiological and psychological mechanisms" [6]. The placebo and nocebo response refers to the wide range of changes in health condition resulting from the administration of an inactive treatment, including spontaneous remission or regression to the mean [6].

The nocebo effect is an important factor in clinical contexts. It contributes to worsening of physiological symptoms and to the formation of a variety of adverse treatment effects which may affect medical adherence and, ultimately, influence medical outcomes [1,2,5,7]. One of the most studied types of nocebo effect is nocebo hyperalgesia, which manifests itself as an increase in pain after implementation of a non-hyperalgesic procedure or substance, i.e. placebo.

Despite the clinical significance of the nocebo effect, its mechanisms have not yet been thoroughly investigated. Much more is known about the mechanisms of the placebo effect [8]; however, studies suggest that the nocebo effect is not just the negative counterpart of the placebo effect [9–11], and the mechanisms underlying these two phenomena may differ [9]. Thus, the results of research on the placebo effect should not be used without prior verification to explain the nocebo effect.

Expectancy, defined as a “conscious, conceptual belief about the future occurrence of an event” [12] is indicated as a key mechanism of placebo effects [13–15]. According to this theoretical approach, all methods of inducing placebo effects, i.e. classical conditioning, verbal suggestions and observational learning, are mediated by expectancy [15]. The term ‘expectancy’ has even been included in definitions of the nocebo effect [12,16] since the early definition that was formulated by Hahn [17]. According to this approach, negative outcomes, which are known as the nocebo effect, are produced by negative expectancies.

At the neural level, it has been shown that negative expectancies and subsequent pain enhancement are associated with an increase in brain activity observed in cortical regions such as insula and anterior cingulate cortex (ACC) [18]. The increase in neurophysiological responses under nocebo treatment has been detected using low alpha EEG and startle reflex techniques [19]. Nevertheless, not only the brain circuits are involved in the nocebo effect but also nociceptive filtering at the spinal level [20]. Moreover, studies conducted on animals and humans have shown that nocebo hyperalgesic effect can be blocked by proglumide, a selective agonist of cholecystokinin (CCK) type-A/B receptors. It thus seems that the nocebo effect has strong biological underpinnings [19].

There is evidence that verbally induced expectancies may result in nocebo hyperalgesia [9,14,21,22]. The results of a few studies suggest that expectancies may be also involved in nocebo hyperalgesia induced by classical conditioning [23–25]. However, in these studies a combined procedure comprising classical conditioning and verbal suggestion of hyperalgesia was used to induce hyperalgesic response. Thus, a definite answer to the question of whether nocebo hyperalgesia induced by conditioning is mediated by expectancies is not possible on that basis.

Although the results of a few studies suggest that conscious processes do not necessarily have to be involved in the classically conditioned nocebo effect [26–33], only in some of them were self-reported expectancies controlled for and measured trial by trial [26,27]. Thus, further research is needed to establish the role of conscious processes in shaping classically conditioned nocebo hyperalgesia [34].

To verify whether expectancies are involved in nocebo hyperalgesia, two conditioning procedures (hidden and open) were compared in this study. In each of these two procedures, participants were provided with the same pain experiences, but the information provided to the participants differed. While participants who had undergone hidden conditioning were not informed about the relationship between conditioned and unconditioned stimuli, those subjected to open conditioning were explicitly informed about it. Conscious expectancies induced in this way were then withdrawn (by informing participants that associations between stimuli would no longer be in effect), so as to be sure that the nocebo hyperalgesia induced as a result of this procedure would not be produced by these expectancies. Thus, investigating hidden and open conditioning could be a way to disentangle conditioning and expectancies and could answer the question about the role of conscious processes in the formation of nocebo hyperalgesia.

It should be noted here that there is a distinction between hidden-open conditioning and hidden-open treatment. Open conditioning requires informing participants about the relationship between conditioned and unconditioned stimuli, while hidden conditioning does not. Hidden-open treatment requires either informing patients or not that they are undergoing medical treatment [35,36].

So far only four studies have used both open and hidden conditioning to induce placebo analgesia [37–40]; these studies showed that hidden conditioning produces significant placebo analgesia, whereas open conditioning elicits a lesser or no effect. The current study is the first to compare hyperalgesic effects induced by hidden and open conditioning. We hypothesized that 1) hidden rather than open conditioning would produce the nocebo effect, 2) the nocebo effect induced by hidden conditioning would be predicted by expectancy.

Another goal of this study was to establish the role of fear in shaping nocebo hyperalgesia induced by hidden and open conditioning. The results of previous studies showed that fear of pain, i.e. a relatively stable trait indicating how fearful individuals are of painful stimulation (hereafter referred to as fear of pain) [41,42] as well as fear of an upcoming painful stimulus, i.e. fear as a state (hereafter referred to as fear) [43], reduces placebo analgesia induced by verbal suggestions. Although one recent study found that fear predicted placebo analgesia induced by hidden conditioning [37], another did not confirm that fear predicted either placebo analgesia or nocebo hyperalgesia induced by hidden conditioning [26]. It has also been found that fear of pain does not predict classically induced placebo analgesia [37] and may not be involved in nocebo hyperalgesia [27,28]. To conclude, the data concerning the effect of dispositional fear of pain on placebo effects are inconclusive; moreover, they mainly come from correlational studies focused on placebo analgesia. As we aimed to search for causal relationships, in the current study participants with a high or low level of dispositional fear of pain were selected to examine the influence of fear of pain on classically induced nocebo hyperalgesia. Since previous studies suggest that fear rather than fear of pain may increase the pain experience, we hypothesized that fear rather than fear of pain would predict nocebo hyperalgesia induced by hidden conditioning.

## Material and methods

Similarly designed screening procedure and experimental sessions have been used in two of our previous studies [26,37]. The description of the study design in this paper follows closely the description of the study design from the previous research in which analogous procedures, i.e. hidden and open conditioning were used to induce the placebo effect [37].

### Participants

A total of 87 females (mean age = 23.84 ± 3.20; range = 19–35 years) participated in the study. Participation in the study was voluntary and financially rewarded. All volunteers were subjected to a screening procedure before the beginning of the experiment. They answered a series of questions concerning their health status. Also, they completed The Hospital Anxiety and Depression Scale (HADS) [44], which allowed to determine the presence of emotional disorders. Data obtained from the screening procedure was used to exclude from the study those individuals who: 1) suffered from pain during last month, 2) took painkillers, 3) regularly took prescribed medications or illegal drugs, 4) overused alcohol or tobacco, 5) have ever had any neurological, respiratory, circulatory, musculoskeletal, metabolic and/or psychiatric disorders, 5) manifested symptoms of anxiety and/or depression. In addition, volunteers completed The Fear of Pain Questionnaire (FPQ-III) [45]. On the basis of previous studies, the mean score and standard deviations of FPQ-III were established [37,43,46,47]. Only those volunteers who had a score higher than one standard deviation above the mean and those who had a score lower than one standard deviation from the mean were included in the study. Those with a medium level of fear of pain (scores 71.10 ± 9.12) were excluded from the experiment. Participants with high and low levels of fear were randomly assigned to each of the three groups, i.e. hidden conditioning, open conditioning and control group. Only participants aged 18 to 35 who did not participate in the pain studies previously were enrolled in the experiment.

Participants were informed that the study was aimed to examine the responses to pain caused by electrical stimulation and that they would receive a series of electrical stimuli during the experiment. The participants were aware that they could withdraw from participation at any time. All participants gave their informed written consent to participate in the experiment. The experimental procedure was conducted in accordance with the principles contained in the Declaration of Helsinki and was approved by the Research Ethics Committee at the Institute of Psychology of the Jagiellonian University.

### Sample size

The sample size was established based on the results from our previous study on classically induced nocebo hyperalgesia [26]. It was decided to examine at least 24 subjects per group because this number covers both planned within-group comparison (fear subgroups with 12 subjects) and planned comparison aimed to compare experimental group to the control group (14 per group). Calculation was performed using effect size *d* of 0.92 and 0.99, respectively. Power calculation was performed *a priori* for within- and between-group planned-comparisons aimed to verify the main hypothesis. Calculation was performed by G*Power (G*Power 3.1.9.2 statistical software [48]) with the alpha level set at 0.05 and 80% power.

### Stimuli

The Constant Current High Voltage Stimulator (Digitimer, Welwyn Garden City, England, Model DS7AH) was used to produce square pulses with a duration of 200 μs. Electrical stimuli were delivered to participant’s non-dominant forearm through two durable stainless steel-disk electrodes 8 mm in diameter with 30 mm spacing. Stimuli at two different intensities were used in the course of experiment. The stimulus at intensity of 2.2T mA (where T is an individual pain threshold) was paired with nocebo stimulus, while the stimulus at intensity 1.5T mA was paired with control stimulus. Both coefficients (2.2 and 1.5) were set based on the results of the preliminary study. They were also used in the previous study [27].

Two color stimuli (blue and orange) were used in this study. Color stimuli were displayed on the screen (17", resolution 1280 x 1024) placed centrally in front of the participant. One of the two colors served as nocebo stimulus and was presented before electrical stimuli at higher intensity, while the other served as control stimulus and preceded electrical stimuli at lower intensity. The colors were counterbalanced.

### Design and procedure

**Fig 1:**
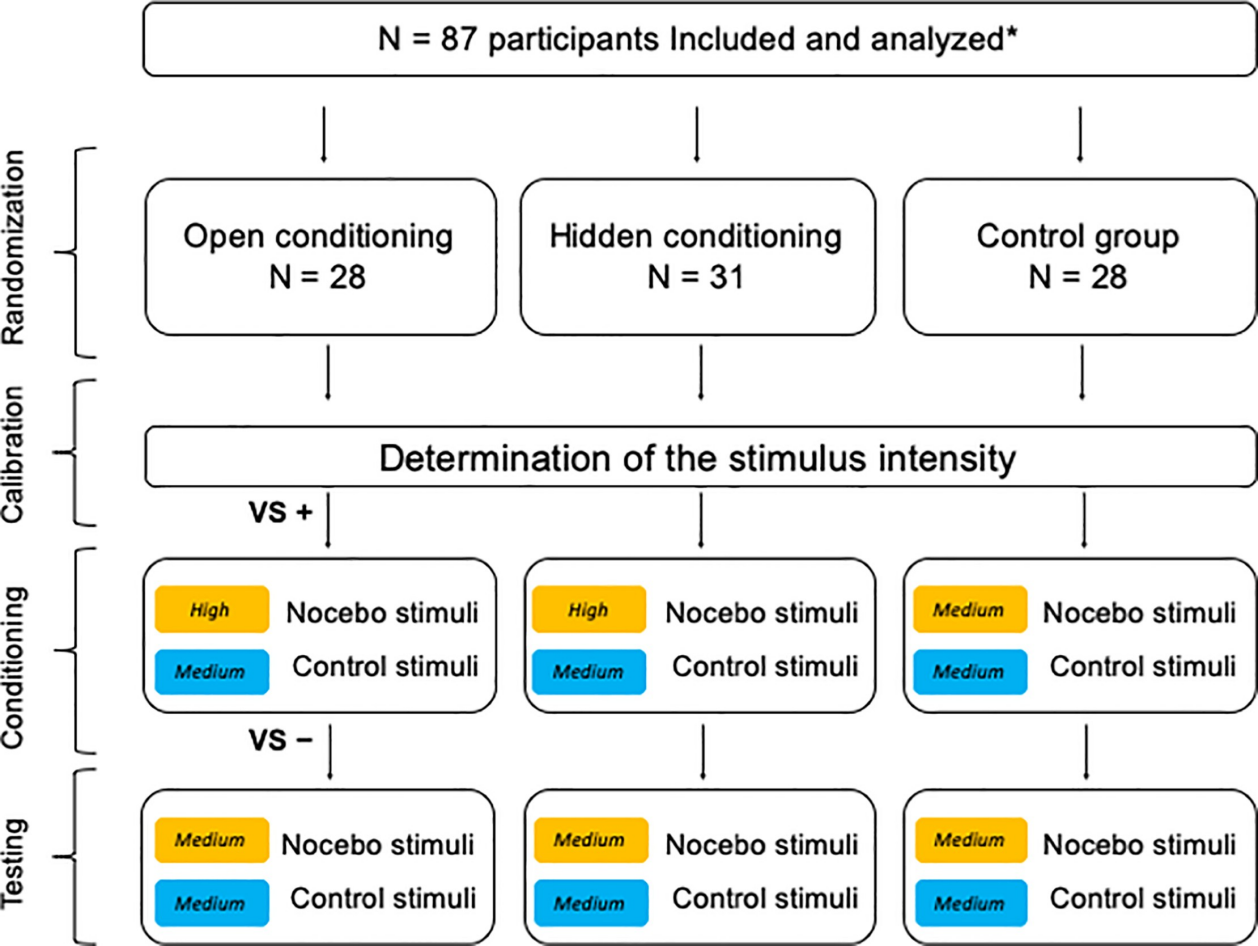
Study design. Participants were randomly assigned to the one of three groups: open conditioning, hidden condition or control group. They received series of electrical stimuli during three successive phases of the experimental session, i.e. calibration, conditioning and testing. Before the conditioning phase, participants in the open conditioning group were informed that one of the colors, e.g. orange (counterbalanced across participants), was related to the higher intensity of electrical stimuli (verbal suggestion, VS+), and before the testing phase they were informed that colors were no longer related to the intensity of the electrical stimuli (VS-). Participants in the hidden conditioning group and control group did not receive any verbal suggestions. Participants in either the hidden conditioning or control group were not informed about any associations between pain and color stimuli, however, the hidden conditioning group received different stimulus levels depending on the color displayed on the screen. *Only participants with low or high fear of pain level were included in the study.

The experimental session consisted of three phases: calibration, conditioning, and testing (Fig 1).

#### Calibration phase

During the calibration phase, the participants received two series of ascending electrical stimuli starting from 0 mA. Each subsequent stimulus was delivered every 5 seconds and was increased by 0.5 mA. The intensity of the pain stimulus was increased until the participant reported tactile, nonpainful sensation (t). Then the intensity of the electrical stimulus was increased until the participant reported the sensation caused by this stimulus as painful. In this way the tactile threshold (t) and pain threshold (T) were determined twice. The average T value was used to calculate the intensity of the pain stimulus that was to be paired with the nocebo stimulus (2.2 T mA), as well as the pain stimulus that was to be paired with the control stimulus (1.5 T mA).

#### Conditioning phase

The conditioning phase started after a five-minute break. During this phase of the experiment, the participants received 72 electrical stimuli. The stimuli were delivered in 4 blocks of 18 stimuli each with a 2-minute break between blocks.

Participants in the hidden and open conditioning groups received pain stimuli at two different intensities, i.e. 1.5T mA and 2.2T mA. The pain stimuli were delivered in the pseudorandom order and were preceded by color stimuli. One of the two colors was presented before more painful stimuli (nocebo stimulus) and the other was displayed before less painful stimuli (control stimulus). Participants rated the intensity of the experienced pain, the expectancy of pain intensity and fear of upcoming pain. They did not provide ratings every time the pain stimulus was delivered. They were asked to rate their experiences only during the second and fourth blocks of conditioning. It aimed to focus their attention on pain sensations rather than pain assessment.

**Fig 2:**
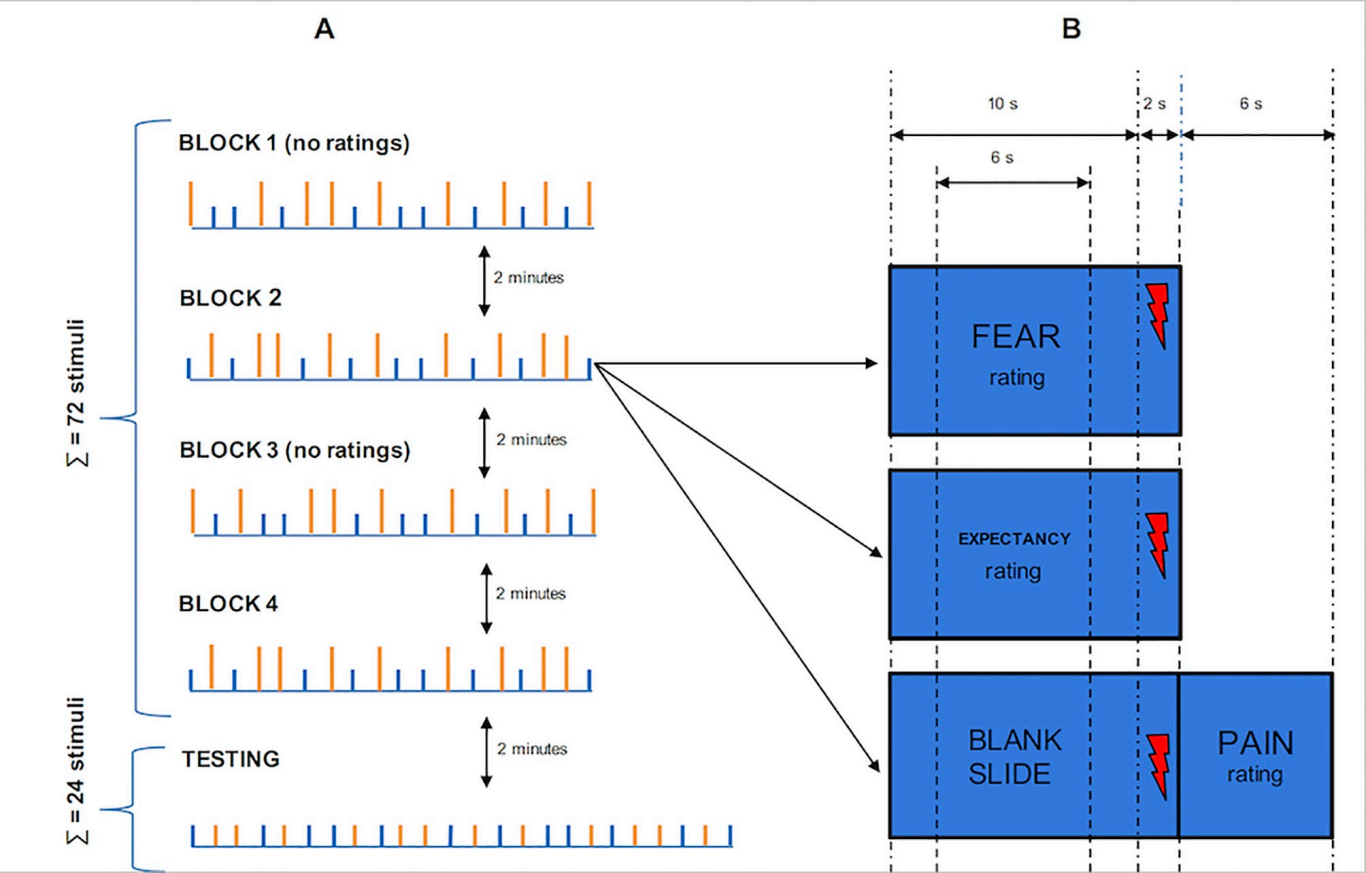
Study design for the hidden conditioning group (example where orange color stimuli serve as nocebos). Part ‘A’ refers to the course of the procedure: there were four blocks of conditioning trials, two of which consisted only of color stimuli without any additional instructions (block 1 and 3). After conditioning, one testing block was applied. Each conditioning block consisted of the application of 18 pain stimuli, whereas the testing block was comprised of 24 pain stimuli. Orange stimuli (orange vertical bars) served as nocebos (painful intensity, i.e. 2.2T mA), while blue stimuli served as control stimuli (moderate intensity, i.e. 1.5T mA). During the testing phase, the applied stimuli were of the same moderate intensity (i.e. 1.5T mA), regardless of the color stimuli. Part ‘B’ refers to the single trial design. Color stimuli were presented for 10 seconds. Note that color stimuli were presented in three different conditions: 1. with the NRS for fear rating; 2. with the NRS for expectancy rating; and 3. without any additional instruction before, but with the NRS for pain intensity rating just after the application of the pain stimulus. Each type of the NRS was presented for 6 seconds, which is the NRS for pain, fear and expectancy. Note that after the presentation of the color stimuli was completed, a pain stimulus of 200 μs duration (depicted by red lightning) was applied.

For each of the two blocks, one-third of the stimuli were presented with the scales for the pain intensity rating, one-third with the scales for the expectancy of pain intensity rating, and one-third with the scales for the fear rating. Each scale was displayed for six seconds during the presentation of the color stimulus that was displayed also during the application of pain stimulus. The scales for the expectancy of pain and fear were displayed before pain stimuli, whereas the scales for the pain intensity were displayed after pain stimuli (Fig 2).

The conditioning procedure was the same in the hidden conditioning group and the open conditioning group. The only difference between these two groups was that the participants in the open conditioning group were informed which of the two colors would precede the less intense pain stimuli and which would be presented before the more intense pain stimuli.

Participants in the control group received a series of electrical stimuli at the same intensity 1.5T mA. Before each of the stimuli one of the two colors (blue or orange) was presented. The repeated application of the stimulus at the same intensity allowed to control for the effects of non-associative learning (sensitization and habituation) and the effects of colors on pain perception [49]. Similarly designed control groups have been used in previous studies in which placebo analgesia or nocebo hyperalgesia were induced by classical conditioning [9,27,37,50].

#### Testing phase

The testing phase began two minutes after the conditioning phase was completed. Participants received 24 electrical stimuli at the same intensity (1.5 mA) preceded by two color stimuli presented in a pseudorandom sequence. Half of the electrical stimuli was preceded by orange color and the remaining half was preceded by blue color. Participants rated intensity of pain, expectancy of pain intensity and fear. The rating scales were displayed exactly as in the conditioning phase. The pain intensity was rated 12 times, while the expectancy of pain and fear were rated 6 times.

At the beginning of the testing phase, participants in the open conditioning group were informed that previously established relationship between the colors and the intensity of pain stimuli would no longer be in effect. Although the colors would be still displayed before the pain stimuli, they would no longer indicate how intense the upcoming pain stimuli would be. This information aimed to rule out expectancies that might have been induced in the open conditioning group. Participants in the hidden conditioning group and the control group did not receive any additional information.

### Measures

The pain intensity, expectancy of pain intensity and fear were measured by means of an 11-point numeric rating scale (NRS). Pain intensity and expectancy of pain intensity were rated on the scale ranged from 0 = ‘no pain’ to 10 = ‘the most pain that is tolerable’. Fear was rated on the scale ranged from 0 = ‘not at all’ to 10 = ‘very much’ (the instruction for fear ratings was: “How much are you afraid of the next pain stimulus”).

The Fear of Pain Questionnaire (FPQ-III) [45] was used to measure the general level of fear associated with pain. After the testing phase was completed, participants were asked questions to determine: 1) whether they had figured out the aim of the study, 2) whether they find out the link between presented colors and pain intensity. After the completion of the study, all participants were fully debriefed.

### Statistical analysis

Baseline differences in age, pain threshold, tactile threshold, height, and body mass were analyzed by one-way analysis of variance (ANOVA) with experimental group as a between-subject factor. To control for differences in pain, pain intensity ratings from the conditioning phase of the study were compared using a repeated-measures analysis of variance (ANOVA) design, with experimental group (hidden conditioning, open conditioning, and control group) as a between-subject factor and condition (nocebo and control) as a within-subject factor. The *F*-tests were followed by *post-hoc* comparisons for the manipulation check. Differences between nocebo- and control-associated pain ratings (hidden and open conditioning groups); blue-control- and orange-control-associated pain ratings (control group) were tested with *post-hoc* Tukey tests. One additional repeated-measures ANOVA was performed to check whether there was an expectancy change between the conditioning and testing phase of the study. Difference in expectancy (nocebo vs. control) was entered as a dependent variable with phase (conditioning, testing) as a within-subjects factor and group as a between-subjects factor (hidden conditioning, open conditioning, and control group). Post-hoc tests were applied in case of statistically significant main or interaction effects.

In order to verify the hypotheses, data from the testing phase of the study were analyzed. Statistical comparisons were performed on pain ratings using a repeated-measures mixed ANOVA design, with experimental group (hidden conditioning, open conditioning, and control group) and fear of pain (high and low) as between-subject factors and condition (nocebo and control) as a within-subject factor. The *F*-tests were followed by within-group planned comparison tests: nocebo- versus control-associated pain ratings in the (1) hidden and (2) open conditioning group, and blue-control- versus orange-control-associated ratings in the (3) control group.

In the next step of the analyses, three between-group planned-comparison tests were performed. (1) To determine whether nocebo hyperalgesia was induced in the hidden conditioning group, the mean difference between nocebo- and control-associated pain ratings from the hidden conditioning group was compared to the mean difference between the two control-associated pain ratings (blue and orange) from the control group. (2) Similarly, to determine whether nocebo hyperalgesia was induced in the open conditioning group, the mean difference between nocebo- and control-associated pain ratings from the open conditioning group was compared to the mean difference between two control-associated pain ratings (blue and orange) from the control group. (3) To test whether the magnitude of nocebo hyperalgesia is greater in the hidden conditioning group than in the open conditioning group, the mean difference between nocebo- and control-associated pain ratings from the hidden conditioning group was compared to the mean difference between nocebo- and control-associated pain ratings from the open conditioning group.

To better characterize the observed effects, Spearman correlation coefficients (*r*) were calculated to determine the degree to which the elicited nocebo hyperalgesia is correlated with either expectancy of pain intensity or fear. To do this, the mean difference between the nocebo- and control-associated pain ratings in the testing phase (i.e. nocebo hyperalgesia) was correlated with the mean differences between the nocebo- and control-associated expectancy of pain intensity and fear ratings. An independent samples Student *t* test was applied to compare the magnitudes of these differences between subgroups of participants, i.e. participants who did or did not declare that there was a relation between the color stimulus and pain intensity.

All the analyses were conducted using STATISTICA data analysis software, version 12 (StatSoft Inc., Tulsa, OK, USA). The level of significance was set at *p* < 0.05; the distribution of data was screened for normality by the Kolmogorov-Smirnov test.

## Results

The characteristics of the participants in each experimental group are presented in Table 1. No significant differences were observed in participant characteristics across the three groups (Table 1): age (*F*_(2,84)_ = 0.68; *p* = 0.51; *ŋ*^2^ = 0.02), pain threshold (*F*_(2,84)_ = 0.83; *p* = 0.43; *ŋ*^2^ = 0.02), tactile threshold (*F*_(2,84)_ = 0.05; *p* = 0.95; *ŋ*^2^ = 0.00), height (*F*_(2,84)_ = 0.36; *p* = 0.70; *ŋ*^2^ = 0.01), and body mass (*F*_(2,84)_ = 1.07; *p* = 0.35; *ŋ*^2^ = 0.02). The descriptive statistics for all the analyzed variables are presented in Table 2. For our primary outcome, i.e. pain ratings associated with control and nocebo stimuli, data were normally distributed which was tested by visual inspection of the histograms and Kolmogorov-Smirnov test results. In each of the group pain ratings were normally distributed (*p* > 0.20).

**Table 1:** Descriptive statistics of each group: Mean and standard deviations.

Experimental group	Fear	N	t (mA)	T (mA)	Age (years)	Height (cm)	Weight (kg)	BMI	FPQ (score)
Control group	-	28	2.21 (0.73)	16.98 (18.86)	23.35 (2.86)	166.00 (5.27)	60.77 (10.16)	23.05	72.00 (24.63)
	low	14	2.33 (1.26)	11.82 (10.57)	23.93 (2.46)	167.5 (4.69)	63.64 (11.04)	22.68	50.36 (7.67)
	high	14	2.11 (0.77)	13.71 (10.32)	24.71 (3.54)	164.71 (6.29)	57.86 (6.16)	21.32	91.00 (11.78)
Hidden conditioning	-	31	2.28 (1.02)	13.56 (7.85)	23.93 (3.70)	167.14 (6.05)	64.21 (11.40)	22.98	74.36 (25.03)
	low	15	2.38 (0.75)	21.88 (24.47)	23.27 (1.94)	166.60 (4.69)	63.27 (10.63)	22.80	48.33 (5.54)
	high	16	2.05 (0.70)	12.39 (10.29)	23.44 (3.58)	165.44 (5.85)	58.43 (9.42)	21.35	94.19 (9.99)
Open conditioning	-	28	2.22 (1.03)	12.77 (10.29)	24.32 (3.02)	166.11 (5.63)	60.75 (9.25)	22.02	70.68 (22.88)
	low	13	2.34 (0.92	15.73 (9.10)	23.54 (2.93)	168.00 (5.87)	63.00 (10.68)	22.32	49.31 (8.55)
	high	15	2.23 (1.14)	11.68 (6.29)	24.27 (4.33)	166.40 (6.30)	65.27 (12.26)	23.57	96.07 (7.62)

**Abbreviations:** N, number of subjects in each experimental group; BMI, Body mass index; FPQ-III, Fear of Pain Questionnaire—III.

**Table 2:** Pain ratings among examined groups: Mean and standard deviations.

Group	N	Nocebo stimuli	Control stimuli	Difference
Hidden conditioning	31	2.13 (1.36)	1.93 (1.22)	0.20 (0.39)
Low fear of pain	15	1.84 (1.26)	1.62 (1.07)	0.22 (0.33)
High fear of pain	16	2.40 (1.44)	2.22 (1.32)	0.18 (0.45)
Open conditioning	28	1.83 (1.12)	1.84 (1.09)	- 0.01 (0.32)
Low fear of pain	13	1.31 (0.87)	1.33 (0.89)	- 0.03 (0.36)
High fear of pain	15	2.28 (1.14)	2.28 (1.08)	0.00 (0.30)
Control group	28	2.76 (1.71)	2.81 (1.80)	- 0.05 (0.35)
Low fear of pain	14	2.45 (1.50)	2.49 (1.59)	- 0.04 (0.39)
High fear of pain	14	3.06 (1.90)	3.13 (2.00)	- 0.07 (0.32)

### Manipulation check

ANOVA on the pain intensity ratings from the conditioning phase revealed a statistically significant main effect of condition (*F*_(1, 84)_ = 85.07, *p* < 0.001, *ŋ*^2^ = 0.50) and an interaction between experimental group and condition (*F*_(2, 84)_ = 21.88, *p* < 0.001, *ŋ*^2^ = 0.44). No significant main effect of the experimental group was found. *Post-hoc* tests revealed that there were no differences in control-associated pain ratings across experimental groups, indicating that pain produced by control stimuli was rated similarly among experimental groups. However, control stimuli were rated as less painful compared to nocebo stimuli in the open (*p* < 0.001) and hidden conditioning groups (*p* < 0.001), indicating that participants discriminated between more and less painful stimuli in both experimental groups. In the control group, there was no difference in pain ratings associated with one color (e.g., orange) compared to control stimuli associated with another color (e.g., blue).

ANOVA on the difference in expectancy revealed significant main effect of group (*F*_(2, 84)_ = 4.98, *p* < 0.01, *ŋ*^2^ = 0.11) and phase (*F*_(1, 84)_ = 13.67, *p* < 0.001, *ŋ*^2^ = 0.14), and group x phase interaction (*F*_(2, 84)_ = 9.55, *p* < 0.001, *ŋ*^2^ = 0.19). Post-hoc comparisons revealed that expectancy changed significantly in the testing phase compared to the conditioning phase in the open-conditioning group only (*p* < 0.001).

Data from the exit questionnaire revealed that nobody figured out the actual aim of the study. Two (6%) participants from the control and 12 (39%) from the hidden conditioning group declared that the color stimuli were associated with pain intensity. Independent samples Student’s t-test did not reveal statistically significant difference in the magnitude of nocebo hyperalgesia in the hidden conditioning group between participants who declared that the color stimuli were associated with pain intensity and those who declared that the color stimuli were not associated with pain intensity (t _(1,29)_ = 0.10; p = 0.92; d = 0.04). Similar results were obtained for expectancy of pain intensity (t _(1,29)_ = 1.04; p = 0.31; d = 0.39) and fear ratings (t _(1,29)_ = 0.81; p = 0.43; d = 0.31).

### Nocebo hyperalgesia

**Fig 3:**
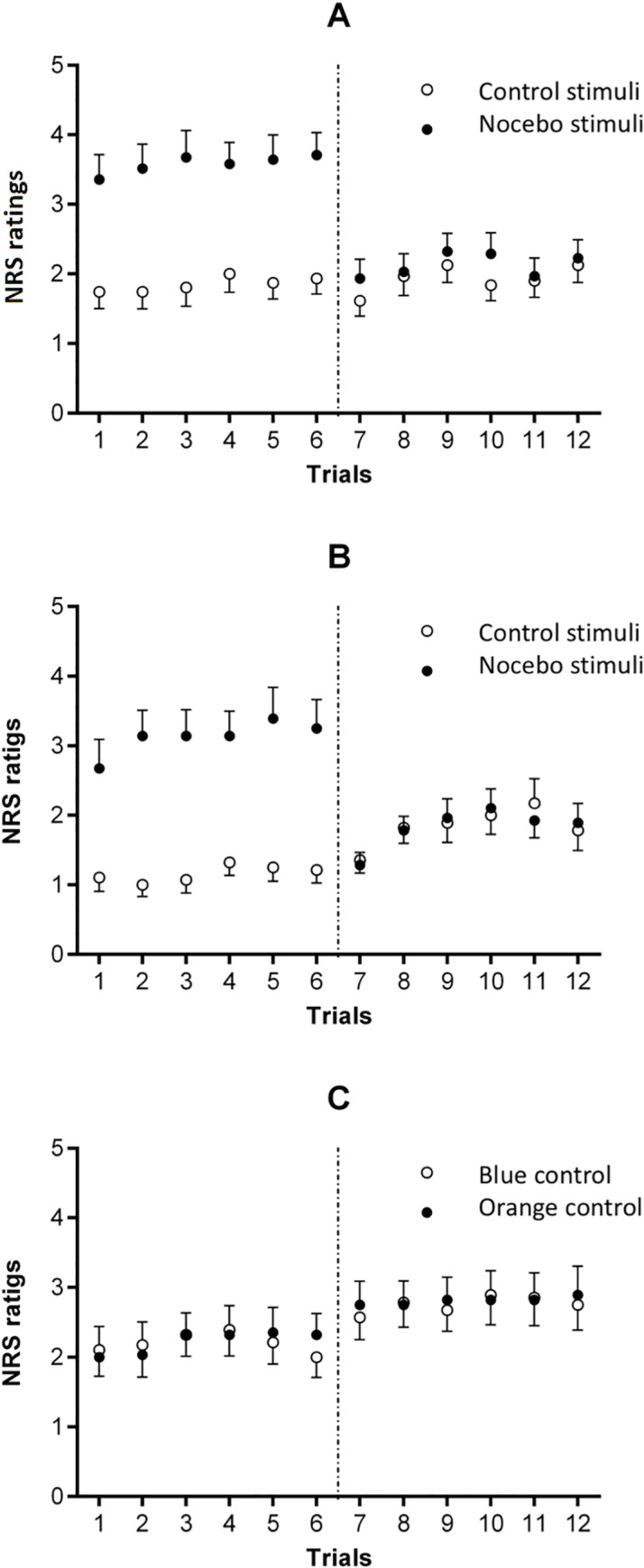
Mean pain intensity ratings during the conditioning and testing phases of the study (separated by vertical dotted lines) in A–the hidden conditioning group (nocebo- *vs*. control-associated ratings), B–the open conditioning group (nocebo- *vs*. control-associated ratings), and C–the control group (orange- *vs*. blue-associated control stimuli). In the conditioning phase, which was divided into 4 blocks, participants rated pain intensity only in one-third of all trials during the second and the fourth block of stimuli. In the testing phase of the study, which consisted of 24 control stimuli, pain intensity was rated 12 times. Error bars represent the SE.

ANOVA on the pain intensity ratings from the testing phase of the study revealed a statistically significant main effect of experimental group (*F*_(2,81)_ = 3.99; *p* = 0.02; *ŋ*^2^ = 0.09), possibly reflecting generally higher pain ratings and sensitization in the control group (Table 2). Main effect of fear of pain (*F*_(1,81)_ = 5.96; *p* = 0.02; *ŋ*^2^ = 0.07), and an interaction between experimental group and condition (*F*_(2,81)_ = 4.19; *p* = 0.02; *ŋ*^2^ = 0.09) were also found. Within-group planned comparisons on the nocebo- versus control-associated pain ratings revealed a statistically significant difference only in the hidden conditioning group (*F*_(1,81)_ = 9.38; *p* = 0.003; *ŋ*^*2*^ = 0.10; Fig 3). Further analysis revealed that this difference was observed only in the low fear of pain subgroup (*F*_(1,81)_ = 5.63; *p* = 0.02; *ŋ*^2^ = 0.06). No difference in the magnitude of the nocebo effect between participants with low and high fear of pain was found (*F*_(1,81)_ = 0.12; *p* = 0.73; *ŋ*^2^ = 0.001).

**Fig 4:**
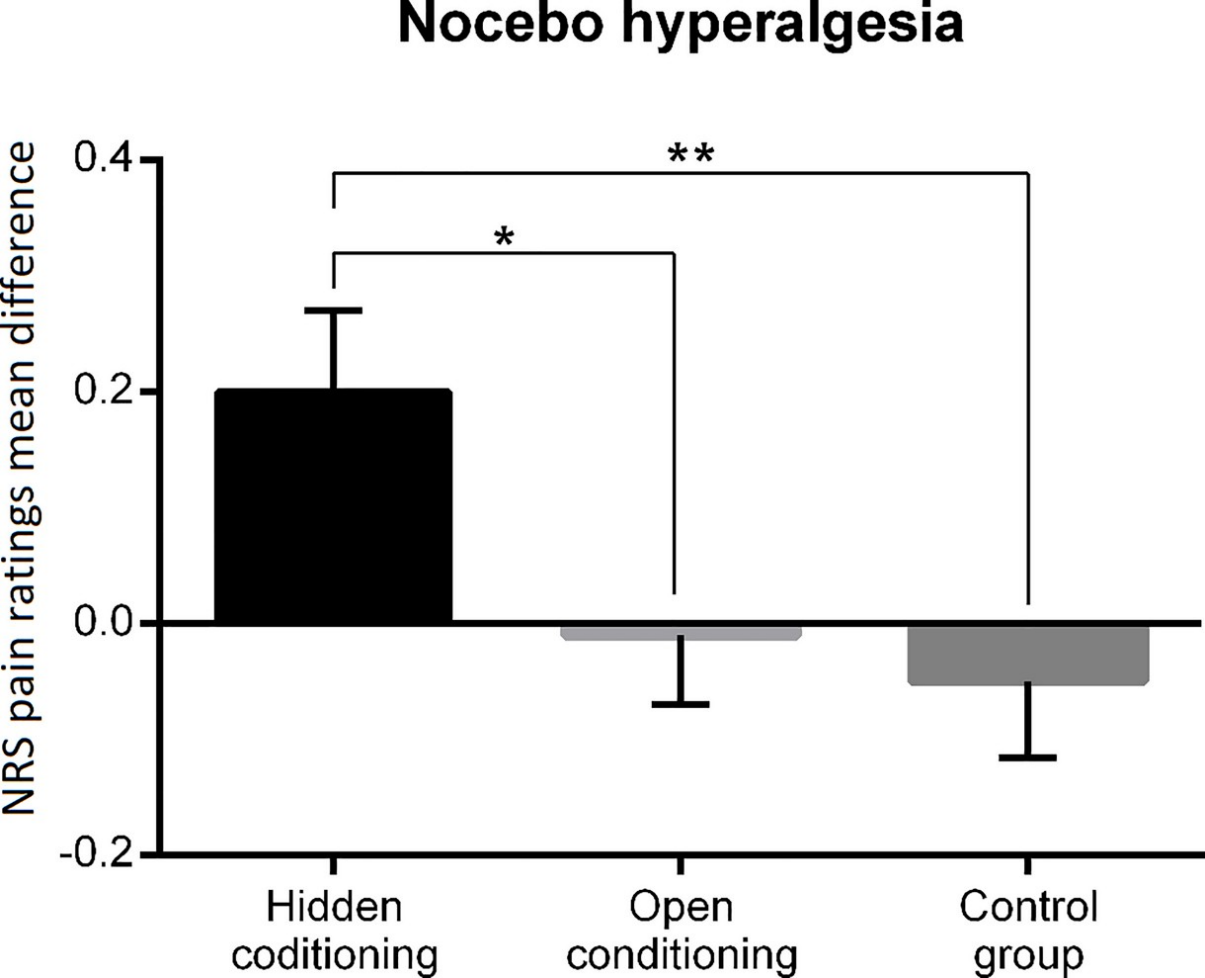
Between-group comparisons of mean pain intensity. Nocebo hyperalgesia was induced only in the hidden conditioning group. The difference between mean pain intensities was significant for both the hidden vs. open conditioning group and hidden conditioning vs. control group. * *p* < 0.05, ** *p* < 0.01. Error bars represent the SE.

Between-group planned comparison on the difference between nocebo- and control-associated pain ratings from the hidden conditioning group compared to the difference between blue-control- and orange-control-associated pain ratings from the control group revealed a statistically significant effect (*F*_(1,81)_ = 7.16; *p* = 0.009; *ŋ*^2^ = 0.08). Subgroup comparisons revealed a trend for significance in both participants with high (*F*_(1,81)_ = 3.50; *p* = 0.06; *ŋ*^2^ = 0.04) and low (*F*_(1,81)_ = 3.66; *p* = 0.06; *ŋ*^2^ = 0.04) fear of pain. Between-group planned comparison on the difference between nocebo- and control-associated pain ratings from the hidden conditioning group compared to the difference from the open conditioning group also revealed a statistically significant effect (*F*_(1,81)_ = 5.03; *p* = 0.03; *ŋ*^2^ = 0.06), indicating that the nocebo hyperalgesia found in the hidden conditioning group was stronger than the effect found in the control group and the open conditioning group (Figs 3 and 4). No significant differences were found within subgroups of high (*F*_(1,81)_ = 1.84; *p* = 0.18; *ŋ*^2^ = 0.02) and low (*F*_(1,81)_ = 3.25; *p* = 0.08; *ŋ*^2^ = 0.04) fear of pain participants. There was also a lack of differences between the control and open conditioning groups (Fig 4).

### Expectancy of pain intensity and fear ratings

The ANOVA on the expectancy of pain intensity ratings from the testing phase of the study did not reveal a statistically significant main effect of experimental group (*F*_(1,81)_ = 1.06; *p* = 0.35; *ŋ*^2^ = 0.03), condition (*F*_(1,81)_ = 2.32; *p* = 0.13; *ŋ*^2^ = 0.03) or an interaction between experimental group and condition (*F*_(2,81)_ = 2.87; *p* = 0.06; *ŋ*^2^ = 0.07). We only found a statistically significant main effect of fear of pain (*F*_(1,81)_ = 10.44; *p* = 0.0018; *ŋ*^2^ = 0.11), which indicates that participants with high fear of pain (measured by FPQ-III) expected stronger pain compared to participants with low fear of pain (Table 3). An analogical pattern of results was observed in the ANOVA on fear of pain ratings. No statistically significant main effect of experimental group (*F*_(2,81)_ = 0.27; *p* = 0.76; *ŋ*^2^ = 0.007), condition (*F*_(1,81)_ = 3.61; *p* = 0.06; *ŋ*^2^ = 0.04) nor an interaction between experimental group and condition (*F*_(2,81)_ = 2.58; *p* = 0.08; *ŋ*^2^ = 0.06) were found. Again, participants with high scores in FPQ-III experienced more fear in the testing phase of the study compared to participants with low FPQ-III scores (*F*_(1,81)_ = 14.01; *p* = 0.0003; *ŋ*^2^ = 0.15) (Table 4). Correlational analyses did not reveal significant relationships between nocebo hyperalgesia and the difference in expectancy of pain intensity and fear ratings (Table 5).

**Table 3:** Expectancy of pain intensity ratings among examined groups: Mean and standard deviations.

Group	N	Nocebo stimuli	Control stimuli	Difference
Hidden conditioning	31	2.54 (1.23)	2.26 (1.14)	0.28 (0.58)
Low fear of pain	15	1.98 (1.01)	1.87 (0.99)	0.11 (0.39)
High fear of pain	16	3.06 (1.21)	2.63 (1.18)	0.44 (0.7)
Open conditioning	28	2.02 (1.46)	2.10 (1.59)	-0.07 (0.69)
Low fear of pain	13	1.21 (0.84)	1.28 (0.83)	-0.08 (0.41)
High fear of pain	15	2.73 (1.53)	2.80 (1.78)	-0.07 (0.87)
Control group	28	2.56 (1.78)	2.49 (1.65)	0.07 (0.34)
Low fear of pain	14	2.31 (1.52)	2.31 (1.56)	0.00 (0.23)
High fear of pain	14	2.81 (2.04)	2.67 (1.77)	0.14 (0.43)

**Table 4:** Fear ratings among examined groups: Mean and standard deviations.

Group	N	Nocebo stimuli	Control stimuli	Difference
Hidden conditioning	31	1.46 (1.30)	1.23 (1.18)	0.24 (0.52)
Low fear of pain	15	0.84 (1.01)	0.64 (0.79)	0.20 (0.37)
High fear of pain	16	2.04 (1.31)	1.77 (1.25)	0.27 (0.65)
Open conditioning	28	1.55 (1.42)	1.27 (1.24)	0.27 (0.93)
Low fear of pain	13	1.00 (1.11)	0.85 (1.08)	0.15 (0.29)
High fear of pain	15	2.02 (1.53)	1.64 (1.29)	0.38 (1.25)
Control group	28	1.51 (1.51)	1.61 (1.63)	-0.10 (0.41)
Low fear of pain	14	1.07 (1.5)	1.07 (1.53)	0.00 (0.29)
High fear of pain	14	1.95 (1.43)	2.14 (1.61)	-0.19 (0.50)

**Table 5:** Correlations between nocebo hyperalgesia, fear and expectancy of pain intensity in each of the groups.

Group	Fear ratings		Expectancy of pain intensity	
	*R*	*p*	*R*	*p*
Hidden conditioning	0.22	0.23	0.19	0.30
Low fear of pain	0.29	0.30	0.16	0.56
High fear of pain	0.21	0.45	0.24	0.37
Open conditioning	0.23	0.25	0.14	0.49
Low fear of pain	0.21	0.48	-0.08	0.80
High fear of pain	0.29	0.30	0.26	0.35
Control group	-0.15	0.44	-0.26	0.18
Low fear of pain	0.00	1.00	-0.29	0.31
High fear of pain	-0.31	0.29	-0.26	0.36

**Abbreviations:** R, Pearson correlation coefficient.

## Discussion

The aim of this study was to use rigorous methods to compare the nocebo hyperalgesic effect induced by open and hidden conditioning procedures and to investigate the influence of expectancy and fear as both a trait and a state on the nocebo effect. We found that the nocebo effect was induced only in the hidden conditioning group. Participants who were not informed about the relationship between pain and color stimuli experienced more pain in relation to placebo stimuli than to control stimuli. We did not find any evidence that expectancy of pain intensity or fear of pain had influenced that effect. Moreover, the data indicate that contingency awareness has not been crucial for the formation of nocebo hyperalgesia.

The results of our study extend current theoretical accounts that emphasize the role of conscious processes in shaping placebo effects [14,15]. According to the model proposed by Colloca and Miller [15], conscious expectancies acquired by decoding information from psychosocial contexts are central to the formation of placebo effects. However, Benedetti and colleagues [14] claim that placebo effects in unconscious processes, such as hormone secretion, may be produced by classical conditioning without the involvement of conscious expectancies. Our comparison of hidden and open conditioning procedures showed that conscious expectancies may not be involved in the nocebo effect, even in the case of conscious physiological process, i.e. pain. Moreover, the results revealed that classical conditioning did not generate consciously accessible expectancies. It has to be noted that the only possible source of expectancies in our hidden conditioning procedure was an experience of the hyperalgesic effect during conditioning since color stimuli were the only placebos used in our study. Also, any conceivable expectancies that could arise were carefully controlled for during the experiment. Our results lead to the conclusion that conscious processes do not necessarily have to be involved in the classically conditioned nocebo effect, which is in line with the results of a few previous studies [24–29] and is consistent with our previous studies, in which the same hidden conditioning procedure was used to induce nocebo hyperalgesia [26,27,34]. The results of our study partially confirmed hypothesis that the nocebo effect may be induced by hidden conditioning; however, effects induced in this way were found to be not predicted by conscious expectancy.

In the open conditioning procedure, direct experience of hyperalgesia was reinforced by information about the manipulation of pain levels during the procedure. Our results showed that only in the open conditioning group relevant pain-related expectancies were induced in the conditioning phase of the study. However, these conscious expectancies were then deliberately withdrawn before the testing phase by informing participants that the colors were no longer associated with the levels of pain stimuli. We have shown that expectancies induced in the conditioning phase were then successfully withdrawn in the testing phase. Moreover, the comparison of participants’ pain-related expectancies in the testing phase in the open conditioning group (where the pain-related suggestion was withdrawn), hidden conditioning group (where the pain-related suggestion was not provided), and the control group has revealed no significant differences. This result shows that we have not only withdrawn previously induced expectancies but we have not produced any new expectancies.

What is more, we did not observe nocebo hyperalgesia in the open conditioning group, which indicates that withdrawal of existing expectancies may prevent the nocebo effect. This is in line with the results of studies showing that classically conditioned associations may be abolished by informing participants that previously learned contingencies are no longer in effect [51–54]. The information about the lack of association between conditioned and unconditioned stimuli that occurred before the testing phase could have eliminated not only conscious expectancies but also consciously inaccessible pre-cognitive associations. Our result is in line with the findings of other studies which failed to induce placebo analgesia [37–40] and nocebo hyperalgesia [21] with open conditioning. Although our study supports the results of the cited studies, there are several methodological differences. First, we used a color as a conditioned stimulus, whereas the previous studies used a cream that could have induced pre-conditioned expectancies [21,38–40]. Second, in our study participants were informed that pain stimulation would no longer be related to placebo versus control stimuli, whereas in the previous studies participants were informed that pain stimulation would be equal [21,38,39]. Third, we used more conditioning trials compared to those studies. Despite these differences, we were unable to elicit the nocebo effect in the open conditioning group.

The results of previous studies on the role of fear and fear of pain in shaping classically induced placebo effects have brought inconclusive results. It has been found that fear may be a predictor of placebo analgesia [37], but other studies did not confirm its role in the formation of placebo analgesia or nocebo hyperalgesia [26,27]. Similarly, inconclusive results were obtained in studies examining whether placebo effects would be predicted by fear of pain. Some previous studies showed that fear of pain was related to placebo analgesia [25], while other studies found that it was involved in neither placebo analgesia [37] nor nocebo hyperalgesia [28]. To establish a causal relationship between fear of pain and the nocebo effect, as well as the differences in hyperalgesic responses in people that differ in the level of fear of pain we divided participants into low and high fear of pain groups. We found the nocebo effect in participants with low and high fear of pain, but there was no difference between both groups. Moreover, fear did not predict nocebo hyperalgesia induced by hidden conditioning, which replicates the findings of our previous studies [26]. Thus, the obtained results did not confirm our hypothesis: they showed that neither fear of pain nor fear are involved in nocebo hyperalgesia induced by classical conditioning.

Interestingly, previous brain imaging studies indicated that fear-related areas such as amygdala and right hippocampus are activated during the exposure to nonconscious nocebo cues [55,56]. Although in our study fear measured at the behavioral level (FPQ-III scores) did not predict nocebo hyperalgesia, activation of these and other structures (for review see [57]) might explain the observed hyperalgesic effect. Furthermore, recent study by Tinnermann et al. [58] has shown that nocebo hyperalgesia depends on the cortico-subcortical-spinal interaction. Whether our paradigm triggers hyperalgesic effect by means of subcortical structures dominantly needs to be addressed in future studies.

There are a few advantages of the current study that should be acknowledged. This is the first study on nocebo hyperalgesia in which the effects of hidden and open conditioning have been compared. In this study, a neutral stimulus, i.e. not connoted medically, was used to induce nocebo hyperalgesia to avoid any pre-conditioned expectancies. The use of color as a placebo allowed us to disentangle treatment expectancies (referring to the treatment effects) from stimulus expectancies (referring to the noxious stimuli), and investigate the role of the latter in shaping nocebo hyperalgesia [59]. Furthermore, the sample size was large compared to the sample sizes of previous studies on the mechanisms of nocebo effects [8,14,21,23,26]. Also, comparison of the hyperalgesic response of participants with high and low dispositional fear allowed us to examine not only the correlational but also the causal relationship between fear of pain and nocebo hyperalgesia.

Some limitations of our study should be also discussed. First, only females participated in the study; in light of sex differences in pain perception [60,61] the results may not be generalizable to men. On the other hand, as only women participated in many of the previous studies, the results of our study can be directly compared to previous research [9,26,37,38,62–65]. Moreover, a number of studies showed that women are overrepresented among patients with chronic pain [66]. Therefore, our results can be applied to the majority of the population that is affected by chronic pain. Second, all the study variables relied on self-reports; however, this is also the case in the other studies on the mechanisms of the nocebo effect [9,14,23,63,67,68]. Third, the effect size in this study is rather small, but this is not something unusual in experimental pain research, especially in the context of inducing placebo or nocebo effects without conscious awareness [28,32]. Fourth, although experimentally induced pain in healthy volunteers seems to be a good model of clinical pain [69], experimental findings might not be directly transferable to clinical settings. Fifth, we have investigated the effect of fear of pain on nocebo hyperalgesia induced by classical conditioning. However, other characteristics (e.g., suggestibility) that may influence placebo effects were not controlled for.

The open-hidden paradigm seems to be a promising way of examining placebo effects. However, it has been used so far in a few studies only. Future research could provide additional evidence of its usefulness in studying the involvement of conscious processes in placebo effects. It would also be advisable to develop more implicit methods for the measurement of expectancies in placebo studies. The use of implicit measurement rather than self-rating would provide further data on the role of expectancy in shaping placebo effects induced by classical conditioning. Moreover, in future experimental studies, it would be valuable to include psychophysiological measures, e.g. EEG or EDA. First, to determine whether conscious or unconscious processes are involved in placebo effects elicited by hidden and open conditioning. Second, to go beyond self-reports and expand the results and conclusions to more objective pain indicators.

Our results have implications for medical practice. They show that the occurrence of nocebo hyperalgesia is not always preceded by conscious expectancies of pain exacerbation. Thus, pain changes can occur even when patients do not anticipate negative outcomes of the treatment. The worsening of patients' condition may result from uncontrollable contextual factors. Identifying such factors, i.e. the conditioned cues that aggravate pain experiences, could be a missing part of modern pain management programs. Explicit forms of invasive and non-surgical treatments emphasize the role of physiological change as a basis for pain treatment. This is not always the case since pain and nociception are two different constructs [70]. Pain is not always congruent with the magnitude of nociception and our study clearly supports this concept and indicates that contextual factors such as visual cues might play a role in that incongruence.

It also seems that identifying patients' baseline characteristics [71], exploring patients' treatment beliefs and prior negative therapeutic history [16,72], should be another important part of pain management programs. It would help to identify those who are at risk of nocebo related effects to take preventive actions. For those patients, special strategies of clinician-patient communication should be developed to minimize the risk of future nocebo-related effects [73].

Nocebo and nocebo-related effects are real phenomena that should be considered in the interpretation of the RCTs. Recent overview of systematic reviews of nocebo effects reported by patients taking placebos in clinical trials has shown that dropouts and adverse effects attributed to nocebo effects are more prevalent in placebo groups compared to untreated groups [74]. Moreover, it seems that the specific treatment effect observed in the RCTs might be underestimated due to nocebo responses [74,75] caused by e.g. negative context or unconscious conditioned stimuli.
